# Predictive value of IBI for acute kidney injury with contrast after PCI in patients with ST-segment elevation myocardial infarction

**DOI:** 10.3389/fcvm.2025.1562731

**Published:** 2025-03-20

**Authors:** Wenjun Ge, Ying Zhang, Song Ge, Mei Chen, Yang Xu

**Affiliations:** ^1^ Department of Cardiology, Suining County People’s Hospital, Suining, Jiangsu, China; ^2^ Department of Radiology, Xuzhou Central Hospital, Xuzhou, Jiangsu, China; ^3^ Department of Geriatric Medicine, Xuzhou Qianghua Hospital, Xuzhou, Jiangsu, China; ^4^ Department of Pathology, Xuzhou Central Hospital, Xuzhou, Jiangsu, China; ^5^ Department of Cardiology, Xuzhou Central Hospital, Xuzhou, Jiangsu, China

**Keywords:** inflammatory burden index, inflammation response, contrast-induced acute kidney injury, ST-segment elevation myocardial infarction, prognosis

## Abstract

**Background:**

Contrast-induced acute kidney injury (CI-AKI) is a common complication in patients with ST-segment elevation myocardial infarction (STEMI) and is associated with an inflammatory response. Inflammatory burden index (IBI) is a novel inflammatory marker, and the relationship between IBI and CI-AKI in STEMI patients is currently unknown. The aim of this study was to investigate the effect of IBI on CI-AKI after percutaneous coronary intervention (PCI) in STEMI patients.

**Methods:**

This was a single-center retrospective observational study consecutively enrolling patients diagnosed with STEMI and successful PCI between August 2022 and December 2024. Logistic regression analysis was used to identify risk factors associated with CI-AKI. Restricted cubic spline (RCS) was used to explore the dose-response relationship between IBI and CI-AKI. The predictive effectiveness of the models was assessed by the net reclassification index (NRI) and the integrated discriminant improvement index (IDI).

**Results:**

A total of 647 patients were included in this study and the incidence of CI-AKI during hospitalization was 78 (12.1%). After adjusting for possible confounding factors, the result showed that IBI > 18.89 (OR = 2.418, 95% CI: 1.331–4.392) was an independent factor for CI-AKI in STEMI patients. RCS results suggested that there was a non-linear dose-response relationship between IBI and CI-AKI. After integrating IBI, the ability of the new model to predict CI-AKI in STEMI patients was significantly improved (NRI = 0.315, IDI = 0.019, *P* < 0.05).

**Conclusion:**

Elevated IBI is an independent risk factor for CI-AKI after PCI in STEMI patients, and there is a non-linear dose-response relationship between IBI and CI-AKI. Integrating IBI can improve the risk stratification of STEMI patients regarding CI-AKI.

## Introduction

With the widespread popularization of reperfusion therapy, the outcomes of patients with ST-segment elevation myocardial infarction (STEMI) have been significantly improved (1). However, a higher survival rate means that more STEMI patients will face long-term chronic disease management, imposing a huge strain and economic burden on the healthcare system (2). In fact, patients with STEMI have many complications. Among them, up to 35% of patients may develop contrast - induced acute kidney injury (CI-AKI) after percutaneous coronary intervention (PCI) (2, 3). Moreover, CI-AKI is associated with poor prognosis, prolonged hospital stays, and increased medical costs (4). Therefore, accurately identifying high-risk STEMI patients has important clinical value.

Inflammatory response plays a crucial role in the development of CI-AKI (4–6). Previous studies have indicated that the neutrophil-to-lymphocyte ratio (NLR) and C-reactive protein (CRP) are important indicators of cardiovascular risk and can be used to enhance risk stratification in patients with various cardiovascular diseases (7). Recently, the inflammatory burden index (IBI), as a new inflammatory marker, has been widely used for risk stratification in cancer patients (8–10). Compared with single inflammatory markers, IBI, calculated from neutrophils, lymphocytes, and CRP, can provide a more stable assessment of inflammation, more accurately reflect the inflammatory state, and predict the prognosis (10–12). In a previous cross-sectional study, Yu et al. found an independent association between IBI levels and the prevalence of CVD, and a significant correlation with the development of cardiovascular diseases (13). In another multicenter study, IBI was proven to be positively associated with the risk of poor outcomes in patients with acute stroke (11). However, the relationship between IBI and CI-AKI in STEMI patients remains unclear. This study aims to explore the predictive value of IBI for CI-AKI after PCI in STEMI patients.

## Methods

### Study population

**Figure 1:**
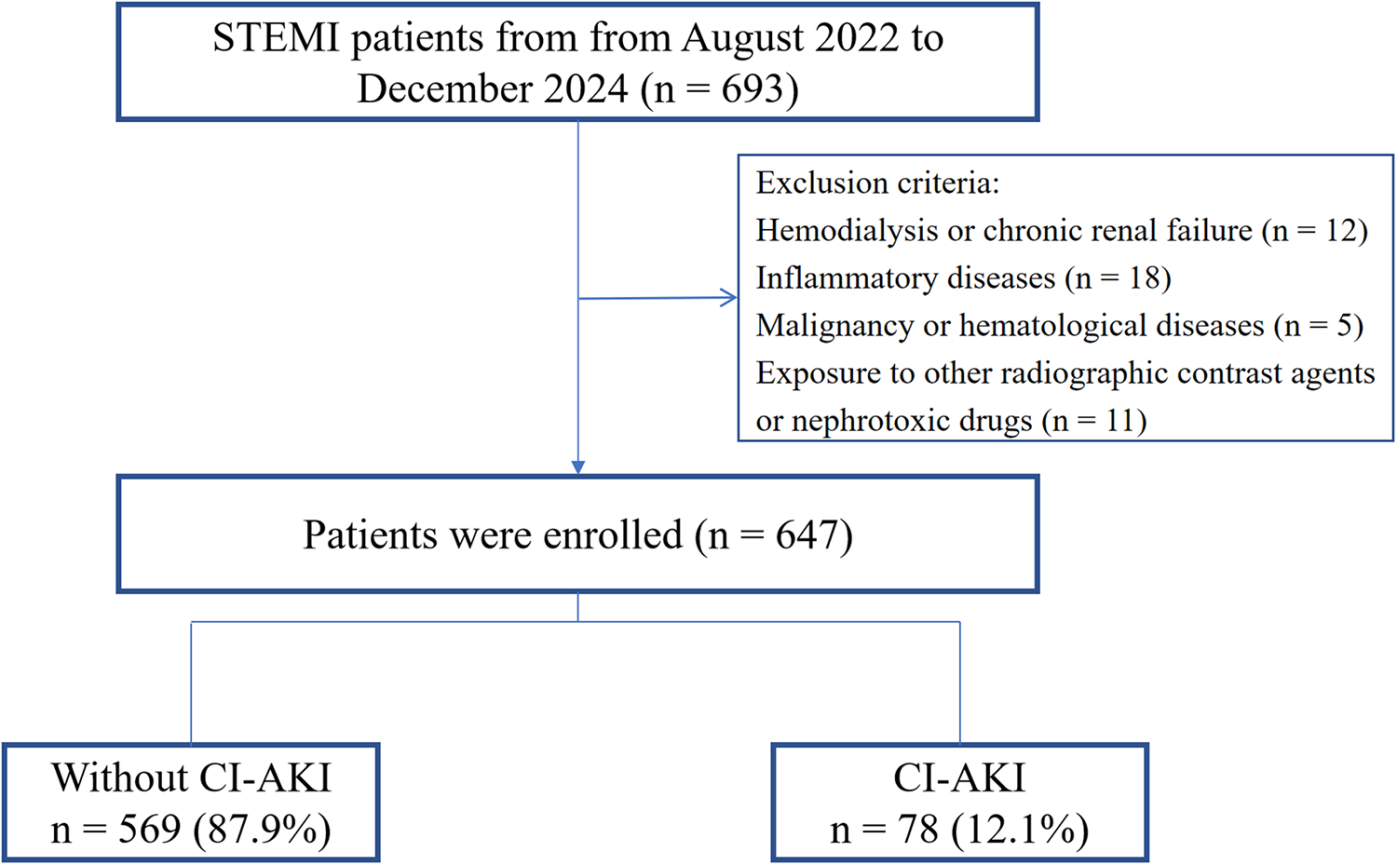
Study flowchart. STEMI, ST-segment elevation myocardial infarction; CI-AKI, contrast-induced acute kidney injury.

This was a single - center retrospective study. We consecutively selected patients diagnosed with STEMI (14) at Suining County People's Hospital from August 2022 to December 2024. Inclusion criteria: (1) Successful primary PCI treatment (TIMI ≥ 2) within 12 h of onset; (2) Complete clinical data. Exclusion criteria: (1) Hemodialysis before admission or chronic renal failure (estimated glomerular filtration rate (eGFR) <30 ml·min^−1^·1.73 m^−2^); (2) Inflammatory diseases; (3) Malignant tumors or hematological diseases; (4) Exposure to other radiocontrast agents or nephrotoxic drugs within 48 h before surgery or within 72 h after surgery. The Institutional Review Board (IRB) of Suining County People's Hospital approved this research protocol (LL-2023-042). According to the relevant regulations of the IRB, since this was a retrospective study and posed no risk to patients, the requirement for signing a written consent form was waived. Finally, 647 patients were included in the study (Figure 1).

### Clinical data collection

Clinical information of all patients was collected according to the medical records, including age, gender, body mass index (BMI), past medical history, and medication use. The serum creatinine (Scr), lymphocyte count, neutrophil count, CRP measured in the emergency room before primary PCI, and the Scr measured 48–72 h after contrast agent exposure were recorded. CI-AKI was defined as an increase in Scr by at least 50% or 0.3 mg/dl within 48–72 h after contrast agent exposure compared to the baseline (15). PLR was defined as the platelet-to-lymphocyte ratio. LCR was defined as the lymphocyte-to-CRP ratio. Systemic immune-inflammation index (SII) was defined as the product of platelet and NLR. Systemic immune-inflammation response index (SIRI) was defined as the product of monocyte and NLR. NLR was defined as the ratio of neutrophil count to lymphocyte count. IBI was defined as the product of CRP and NLR (8–10). In addition, the peak values of troponin I (TNI) and N - terminal pro - B - type natriuretic peptide (NT-proBNP) during hospitalization were also recorded. The recorded medications included aspirin, P_2_Y_12_ inhibitors, β-blockers, statins, nitrates, angiotensin - converting enzyme inhibitors (ACEI) or angiotensin receptor blockers (ARB), and diuretics. In all patients, PCI was performed by the same team who were unaware of the research protocol in accordance with the relevant guideline (1).

### Statistical analysis

All data were statistically analyzed using SPSS (Version 27.0, Chicago, USA) and R 4.3.1. The normality of the data was tested by the Kolmogorov–Smirnov test. Continuous variables conforming to a normal distribution were expressed as mean ± standard deviation and analyzed using the *t*-test. Continuous variables not conforming to a normal distribution were expressed as median (interquartile range) and analyzed using the Mann–Whitney *U*-test. Categorical variables were expressed as counts and percentages and analyzed using the χ^2^-test for statistical analysis. Pearson or Spearman analysis was used to explore the correlations between IBI and other inflammatory factors. According to the variance inflation factor, variables with *P* < 0.05 in the univariate regression analysis were included in the multivariate regression analysis using the stepwise forward method to identify independent risk factors associated with CI-AKI. Restricted cubic splines (RCS) were used to explore the dose-response relationship between IBI and CI-AKI. The predictive performance of the old and new models was evaluated by the receiver operating characteristic curve (ROC), net reclassification index (NRI), and integrated discrimination improvement index (IDI). Analyze the mediating effects between the independent variable IBI, the mediating variables NT-poBNP and FBG, and the dependent variable according to the *Z*-score transformation method. *P* < 0.05 was considered to be significant.

## Results

### Patients characteristics

A total of 647 patients were enrolled in this study. The incidence of CI-AKI during hospitalization was 78/647 (12.1%). Compared with the without CI-AKI group, patients in the CI-AKI group had higher age, neutrophil count, CRP, IBI, NLR, PLR, SII, fasting blood glucose (FBG), TNI, NT-proBNP, and Killip class. The proportions of diabetes and infarct-related arteries (IRA)-left anterior descending artery (LAD) were larger, while the lymphocyte count, eGFR, LCR, and left ventricular ejection fraction (LVEF) were lower. All the differences were statistically significant (*P* < 0.05) (Table 1).

**Table 1:** Patient characteristics.

Variable	Without CI-AKI (*n* = 569)	CI-AKI (*n* = 78)	*P*
Age, years	62.84 ± 13.08	65.56 ± 10.24	0.036
Female, *n* (%)	164 (28.82)	27 (34.62)	0.293
Heart rate, bpm	80.33 ± 14.98	82.50 ± 14.38	0.229
SBP, mmHg	127.09 ± 20.14	126.71 ± 20.29	0.875
DBP, mmHg	78.66 ± 14.01	79.21 ± 14.91	0.748
BMI, kg/m^2^	24.64 ± 3.80	25.42 ± 4.38	0.096
Smoking, *n* (%)	264 (46.40)	37 (47.44)	0.863
Hypertension, *n* (%)	253 (44.46)	34 (43.59)	0.884
Diabetes mellitus, *n* (%)	141 (24.78)	29 (37.18)	0.020
MI, *n* (%)	33 (5.80)	6 (7.69)	0.685
CKD, *n* (%)	17 (2.99)	1 (1.28)	0.623
CABG, *n* (%)	1 (0.18)	0 (0.00)	1.000
IBI	32.22 ± 48.75	62.26 ± 72.62	<.001
NLR	6.73 ± 5.76	9.54 ± 8.00	<.001
LCR	1.22 ± 1.79	0.46 ± 0.63	<.001
PLR	164.85 ± 96.50	205.03 ± 134.31	0.034
SII	1,425.4 ± 1,225.1	2,120.7 ± 2,758.3	<.001
SIRI	3.66 ± 4.47	3.80 ± 2.74	0.431
White blood cell, 10^9^/L	10.32 ± 3.19	10.86 ± 3.32	0.166
Neutrophil, 10^9^/L	8.07 ± 3.97	9.10 ± 3.36	0.028
Lymphocyte, 10^9^/L	1.75 ± 1.22	1.25 ± 0.59	<.001
Hemoglobin, g/L	140.23 ± 16.72	139.69 ± 17.36	0.790
Platelet, 10^9^/L	217.24 ± 59.18	206.85 ± 59.44	0.146
Serum creatinine, μmol/L	66.34 ± 20.22	67.04 ± 19.69	0.774
eGFR, ml/min/1.73 m^2^	103.24 ± 20.66	97.32 ± 18.90	0.017
FBG, mmol/L	6.72 ± 2.77	8.17 ± 3.53	<.001
Total cholesterol, mmol/L	4.30 ± 1.01	4.41 ± 0.85	0.301
Triglycerides, mmol/L	1.53 ± 1.14	1.31 ± 0.67	0.099
HDL-C, mmol/L	0.98 ± 0.25	1.01 ± 0.15	0.132
LDL-C, mmol/L	2.76 ± 0.88	2.86 ± 0.76	0.321
CRP, mg/L	3.23 (0.94, 7.00)	5.54 (2.84, 9.60)	<.001
TnI, ng/ml	21.87 (8.08, 37.14)	27.31 (14.60, 36.03)	0.023
NT-proBNP, pg/ml	1,152.0 (454.2, 2,701.0)	2,840.0 (1,349.8, 4,415.0)	<.001
LVEF, %	52.45 ± 6.82	49.92 ± 6.77	0.002
Killip class, *n* (%)			0.036
Ⅰ	552 (85.32)	493 (86.64)	
Ⅱ	30 (4.64)	26 (4.57)	
Ⅲ	2 (0.31)	2 (0.35)	
Ⅳ	63 (9.74)	48 (8.44)	
IABP, *n* (%)	16 (2.81)	5 (6.41)	0.180
Duration of operation, min	60.27 ± 20.26	62.59 ± 19.15	0.341
Contrast agent, ml	103.32 ± 37.89	110.00 ± 41.97	0.150
IRA-LAD, *n* (%)	271 (47.63)	47 (60.26)	0.036
IRA-LCX, *n* (%)	56 (9.84)	9 (11.54)	0.640
IRA-RCA, *n* (%)	239 (42.00)	22 (28.21)	0.020
IRA-LM, *n* (%)	3 (0.53)	0 (0.00)	1.000
Aspirin, *n* (%)	567 (99.65)	78 (100.00)	1.000
P_2_Y_12_ inhibitor, *n* (%)	568 (99.82)	78 (100.00)	1.000
Statins, *n* (%)	566 (99.47)	78 (100.00)	1.000
ACEI/ARB, *n* (%)	270 (47.45)	44 (56.41)	0.138
β-blockers, *n* (%)	490 (86.12)	67 (85.90)	0.958
Nitrates, *n* (%)	226 (39.72)	25 (32.05)	0.192
Heparin, *n* (%)	473 (83.13)	64 (82.05)	0.812
Diuretics, *n* (%)	309 (54.31)	50 (64.10)	0.103

BMI, body mass index; IRA, infarct-related arteries; IABP, intra-aortic balloon pump; LVEF, left ventricular ejection fraction; CKD, chronic kidney disease; SBP, systolic blood pressure; DBP, diastolic blood pressure; IBI, inflammatory burden index; MI, myocardial infarction; LAD, left anterior descending; LCX, left circumflex artery; RCA, right coronary artery; LM, left main; ACEI, angiotensin-converting-enzyme inhibitor; ARB, angiotensin II receptor blocker; HDL-C, high-density leptin cholesterol; LDL-C, low-density leptin cholesterol; CRP, C-reactive protein; NLR, neutrophil-to-lymphocyte ratio; TnI, troponin I; NT-proBNP, N-terminal pro-B-type natriuretic peptide; FBG, fasting blood glucose; CI-AKI, contrast-induced acute kidney injury; PLR, platelet-to-lymphocyte ratio; LCR, lymphocyte-to-CRP ratio; SII, systemic immune-inflammation index; SIRI, systemic immune-inflammation response index.

### ROC analysis of IBI for CI-AKI

**Figure 2:**
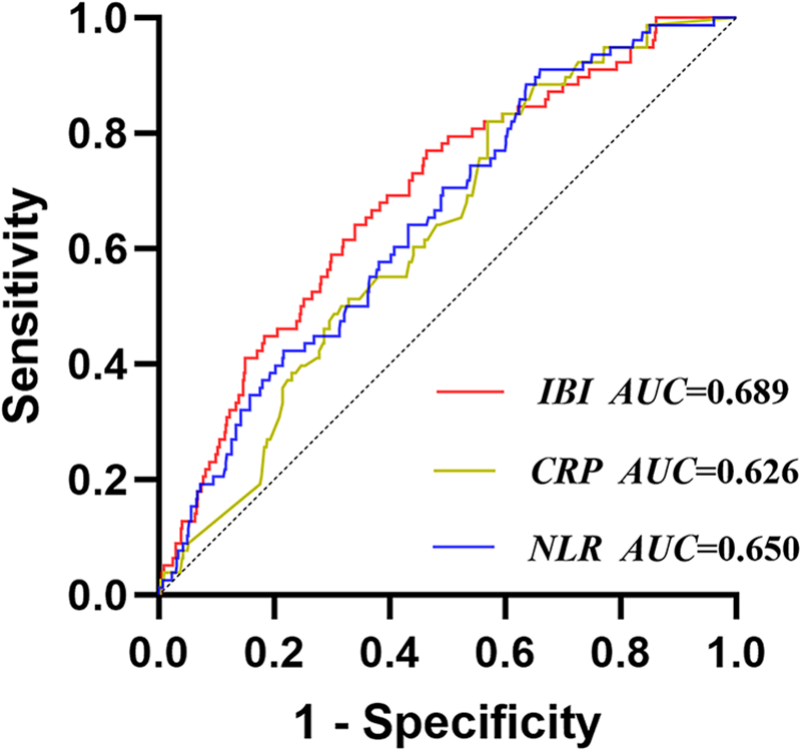
Receiver operating characteristic analysis (ROC) of IBI for CI-AKI. IBI, inflammatory burden index; CRP, C-reactive protein; NLR, neutrophil-to-lymphocyte ratio; CI-AKI, contrast-induced acute kidney injury.

The ROC results showed that the areas under the curves (AUC) of CRP, NLR, and IBI for predicting CI-AKI during hospitalization were 0.626, 0.650, and 0.689 respectively, and the corresponding *P*-values were all less than 0.05, indicating statistical differences. The cut-off value of IBI for predicting CI-AKI was 18.89, with a sensitivity of 76.9% and a specificity of 53.6%. The results of DeLong test showed that the AUC of IBI was significantly higher than that of CRP (*Z* = 3.666, *P* < 0.001), but there was no significant difference between IBI and NLR (*Z* = 1.466, *P* = 0.143) (Supplementary Table S1, Figure 2). The correlation analysis of NLR, lymphocyte, neutrophil, CRP, and IBI was showed in Supplementary Table S2.

**Figure 3:**
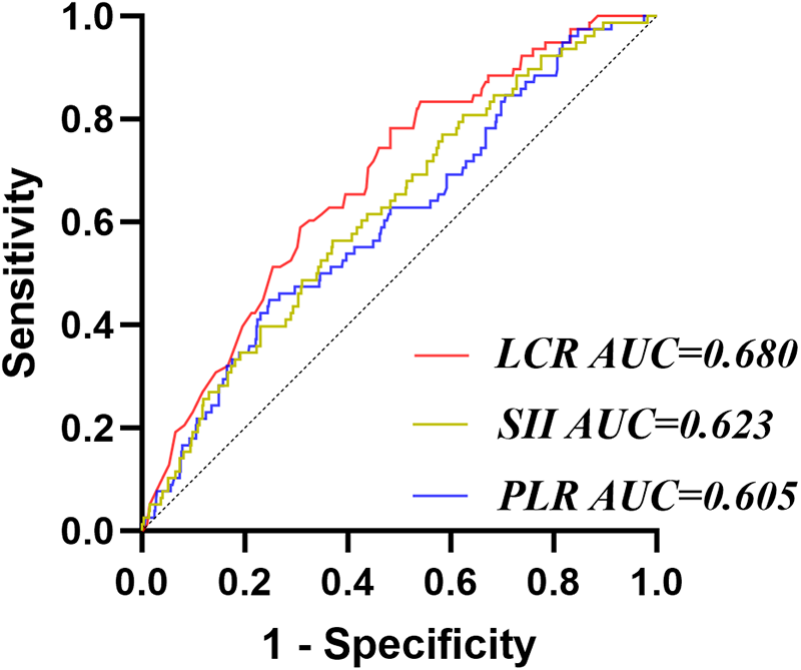
Receiver operating characteristic analysis (ROC) of other inflammatory factors for CI-AKI. PLR, platelet-to-lymphocyte ratio; LCR, lymphocyte-to-CRP ratio; SII, systemic immune-inflammation index.

The ROC results showed that the AUC of PLR, SII, and LCR for predicting CI-AKI were 0.605, 0.623, and 0.680 respectively, and the corresponding *P*-values were all less than 0.05. DeLong test showed that the AUC of IBI was significantly higher than that of PLR (*Z* = 2.570, *P* = 0.010) and SII (*Z* = 2.338, *P* = 0.019), but there was no significant difference between IBI and LCR (*Z* = 1.049, *P* = 0.294) (Figure 3).

### Logistic regression analysis of CI-AKI

**Figure 4:**
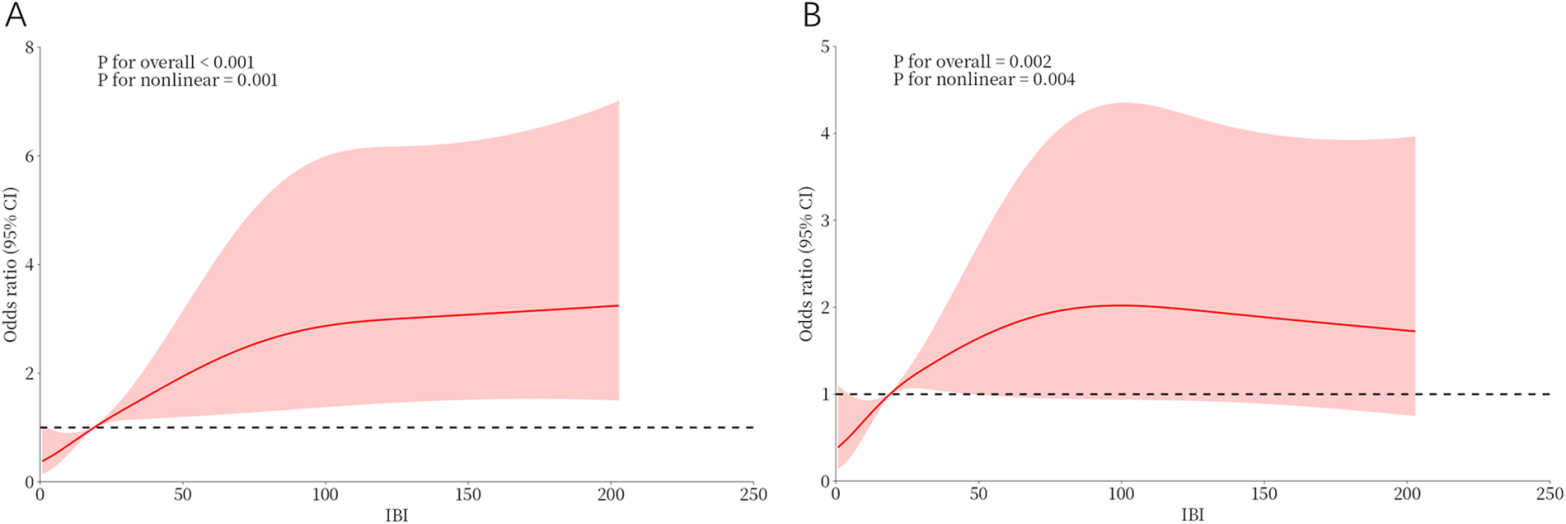
Dose-response relationship between IBI and CI-AKI from RCS analysis. **(A)** Unadjusted dose-response relationship between IBI and CI-AKI; **(B)** Adjusted dose-response relationship between IBI and CI-AKI. IBI, inflammatory burden index; CI-AKI, contrast-induced acute kidney injury.

Univariate logistic regression analysis showed that IBI > 18.89, NLR, lymphocyte count, LVEF, eGFR, CRP, FBG, Killip class >2, IRA- LAD, diabetes, and NT-proBNP were associated with the occurrence of CI-AKI during hospitalization (*P* < 0.05) (Supplementary Table S3). These variables were included in the multivariate logistic regression using the stepwise forward method. The results showed that FBG (OR = 1.098, 95% CI: 1.023–1.179), NT-proBNP (OR = 1.486, 95% CI: 1.189–1.857), lymphocyte (OR = 0.645, 95% CI: 0.439–0.949), and IBI > 18.89 (OR = 2.418, 95% CI: 1.331–4.392) were independent factors affecting the occurrence of CI-AKI in STEMI patients (Table 2). The results of RCS suggested a non-linear dose-response relationship between IBI and CI-AKI, indicating that the higher the IBI, the higher the risk of CI-AKI might be (Figure 4).

**Table 2:** Multivariate regression analysis for CI-AKI.

Variable	OR (95% CI)	*P*
IBI >18.89	2.418 (1.331–4.392)	0.004
Lymphocyte	0.645 (0.439–0.949)	0.026
NT-proBNP	1.486 (1.189–1.857)	<.001
FBG	1.098 (1.023–1.179)	0.010

IBI, inflammatory burden index; NT-proBNP, N-terminal pro-B-type natriuretic peptide; FBG, fasting blood glucose; CI-AKI, contrast-induced acute kidney injury.

### Comparative analysis of the traditional model and the new model

**Figure 5:**
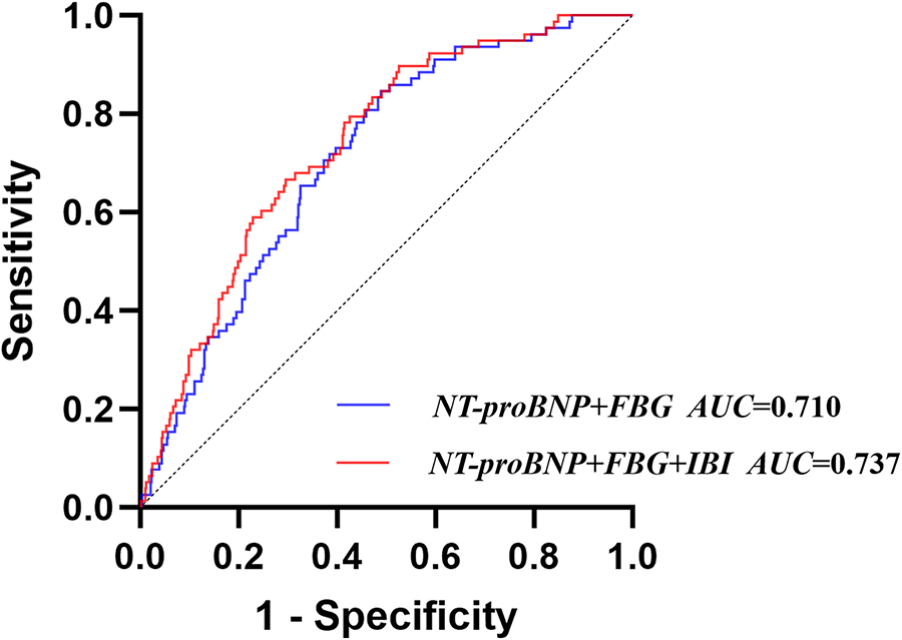
Receiver operating characteristic analysis (ROC) of models for contrast-induced acute kidney injury.

Based on the results of the multivariate logistic regression analysis and previous studies, a traditional model was established by including FBG and NT-proBNP, and a new model was established by integrating IBI. The results showed that the AUC of the traditional model was 0.710 (95% CI: 0.656–0.764), with a sensitivity of 84.6% and a specificity of 51.1%; the AUC of the new model was 0.737 (95% CI: 0.684–0.790), with a sensitivity of 89.7% and a specificity of 47.5%. DeLong test suggested that the AUC of the new model was significantly higher than that of the traditional model (*Z* = 2.308, *P* = 0.021) (Figure 5, Supplementary Table S4). Next, the NRI and IDI of the models were calculated and compared. The results showed that integrating IBI could significantly improve the traditional model for CI-AKI (NRI = 0.315, 95% CI: 0.084–0.546, *P* = 0.007; IDI = 0.019, 95% CI: 0.001–0.037, *P* = 0.036) (Table 3).

**Table 3:** Incremental value of IBI for CI-AKI.

Variable	NRI		IDI	
	Estimate (95% CI)	*P*	Estimate (95% CI)	*P*
NT-proBNP + FBG	Reference	–	Reference	–
NT-proBNP + FBG + IBI	0.315 (0.084–0.546)	0.007	0.019 (0.001–0.037)	0.036

IBI, inflammatory burden index; NT-proBNP, N-terminal pro-B-type natriuretic peptide; FBG, fasting blood glucose; CI-AKI, contrast-induced acute kidney injury.

### The mediating effects of NT-poBNP and FBG

The results showed that there was a significant correlation between IBI and CI-AKI (*c* = 0.007, *SE* = 0.002); after adding FBG and NT-poBNP respectively to the model of IBI and CI-AKI, there was still a significant correlation between IBI and CI-AKI (*c'*_FBG_ = 0.006, *SE* = 0.002; *c'*_BNP_ = 0.006, *SE* = 0.002); there was a significant correlation between IBI and FBG, NT-poBNP (*a*_FBG_ = 0.010, *SE* = 0.002; *a*_BNP_ = 0.005, *SE* = 0.001); there was a significant correlation between FBG, NT-poBNP and CI-AKI (*b*_FBG_ = 0.112, *SE* = 0.035; *b*_BNP_ = 0.409, *SE* = 0.103). Through the mediating effect analysis, it was found that in the association between IBI and CI-AKI, the mediating effects of FBG and NT-poBNP were statistically significant (*Z*_FBG_ = 2.658, *P* = 0.008; *Z*_BNP_ = 3.072, *P* = 0.002). The mediating value corresponding to FBG was 0.0011 (95% CI: 0.0003–0.0019), and the proportion of the mediating effect was 16.0%; the mediating value corresponding to BNP was 0.002 (95% CI: 0.0008–0.0033), and the proportion of the mediating effect was 29.21%. This indicates that IBI can affect the occurrence of CI-AKI by influencing the levels of FBG and NT-poBNP.

## Discussion

To the best of our knowledge, this study is the first to explore the relationship between IBI and CI-AKI in STEMI patients. The main findings are as follows. First, Elevated IBI is an independent risk factor for CI-AKI after PCI in STEMI patients. Second, there is a non-linear dose-response relationship between IBI and CI-AKI after PCI in STEMI patients. Third, integrating IBI can improve the risk stratification of CI-AKI after PCI in STEMI patients.

At present, CI-AKI remains a common complication after PCI in STEMI patients and is also one of the main causes of iatrogenic renal impairment (16). In this study, the incidence of CI - AKI during hospitalization after emergency PCI in STEMI patients was 12.1%. Previous studies have shown that it is closely related to contrast agents, myocardial damage, and insufficient perfusion (4, 15). Given the negative impact of CI - AKI on clinical prognosis, it is necessary to explore more relevant risk factors, so as to optimize risk stratification and improve prognosis.

As is well-known, inflammation is a fundamental process underlying both STEMI and renal impairment (17, 18). Although inflammatory markers such as IL-6, IL-8, and TNF-α have been proven to be sensitive and reliable markers for the risk stratification of CI-AKI (19, 20), these test indicators usually cannot be obtained routinely or rapidly, limiting their application in clinical practice. Complete blood count is a simple and commonly used measurement method that can be rapidly obtained before primary PCI in STEMI patients (5, 21). In recent years, IBI has been widely used for risk stratification in cancer and has been proven to be a better biomarker compared to single inflammatory markers (8–10). In a previous cross-sectional study, Yu et al. found an independent association between IBI levels and the prevalence of CVD, and a significant correlation with the development of cardiovascular diseases (13). In another multicenter study, IBI was proven to be positively associated with the risk of poor outcomes in patients with acute stroke. Compared with other inflammatory markers, IBI had the highest area under the curve and reclassification index (11). However, in STEMI, research on IBI is still in its initial stage, and the relationship between IBI and CI-AKI after PCI in STEMI patients remains unclear. This study found that in STEMI patients, elevated IBI was an independent risk factor for the occurrence of CI-AKI after PCI, and there is a non-linear dose-response relationship between IBI and CI-AKI. As a traditional inflammatory marker, CRP is an important predictor of the onset and prognosis of cardiovascular and cerebrovascular diseases (22, 23). High levels of CRP can mediate the expression of adhesion molecules, reduce the production of NO, impair the antioxidant defense function of endothelial cells, and lead to endothelial dysfunction, which is considered an important factor in the occurrence of CI-AKI (22). Neutrophils and lymphocytes are two types of peripheral inflammatory cells and important components of the immune system, being crucial for the development of inflammation and subsequent pathological processes (24–26). During myocardial infarction, the body is in a stress state, and elevated levels of catecholamines and cortisol lead to a decrease in lymphocyte count and an increase in neutrophil count (27). Previous studies have confirmed that a decrease in peripheral blood lymphocyte count and an increase in neutrophil count in patients with acute chest pain are related to the progression of atherosclerosis, impairment of coronary microcirculation, and the occurrence of major cardiac events (28, 29). By taking advantage of CRP and NLR, IBI analyzes the balance between acute inflammation and immune-mediated inflammation, providing a relatively complete picture of the body's pro-inflammatory and immune states. In addition, the results of the mediating effect analysis show that IBI can affect the occurrence of CI-AKI by influencing the levels of FBG and NT-poBNP. This may partly explain the findings of this study.

Consistent with previous research results (5, 17, 20, 30), NT-proBNP, and FBG were also proven to be independent risk factors for CI-AKI after PCI in STEMI patients in our study. After integrating IBI into the traditional risk model, the predictive ability for CI-AKI after PCI in STEMI patients was significantly improved. IBI combines the advantages of CRP and NLR, comprehensively reflecting the body's inflammatory and immune states. IBI may have a stronger ability in predicting outcome events compared to some other traditional inflammatory markers (CRP, SII, and PLR). Considering the economic and convenient advantages of IBI, using IBI as an auxiliary method for risk stratification of CI-AKI before PCI in STEMI patients and screening high-risk populations may be highly practical.

It should be noted that this study has certain limitations. First, this is a single-center retrospective study, and there may be some inevitable biases in the obtained data. Second, although this study has demonstrated an association between high IBI and the occurrence of CI-AKI after PCI in STEMI patients, the specific mechanism of action may need to be further explored through future basic research. Finally, all the STEMI patients included in this study underwent successful PCI treatment. Therefore, some of the conclusions may need to be replicated and verified in different populations.

### Conclusions

Elevated IBI is an independent risk factor for CI-AKI after PCI in STEMI patients, and there is a non-linear dose-response relationship between IBI and CI-AKI. Integrating IBI can improve the risk stratification of STEMI patients regarding CI-AKI.

## Data Availability

The raw data supporting the conclusions of this article will be made available by the authors, without undue reservation.
