# Supplementary material for: Predictive value of IBI for acute kidney injury with contrast after PCI in patients with ST-segment elevation myocardial infarction
Source: Front Cardiovasc Med. 2025 Mar 20;12:1562731. doi: 10.3389/fcvm.2025.1562731 (PMC11965358; doi:10.3389/fcvm.2025.1562731)
Supplement: Supplementary file 1 [file Table1.docx]

**Supplementary Table 1. ROC Curve for CI-AKI**

|  | AUC | 95% CI | *P* | Cut-off | Sensitivity | Specificity |
| --- | --- | --- | --- | --- | --- | --- |
| IBI | 0.689 | 0.629-0.750 | <0.001 | 18.89 | 0.769 | 0.536 |
| CRP, mg/L | 0.626 | 0.567-0.685 | <0.001 | 2.41 | 0.821 | 0.431 |
| NLR | 0.650 | 0.590-0.711 | <0.001 | 3.98 | 0.885 | 0.366 |

IBI = inflammatory burden index; CRP = C-reactive protein; NLR = neutrophil-to-lymphocyte ratio; CI-AKI = contrast-induced acute kidney injury.
