# Supplementary material for: Predictive value of IBI for acute kidney injury with contrast after PCI in patients with ST-segment elevation myocardial infarction
Source: Front Cardiovasc Med. 2025 Mar 20;12:1562731. doi: 10.3389/fcvm.2025.1562731 (PMC11965358; doi:10.3389/fcvm.2025.1562731)
Supplement: Supplementary file 2 [file Table2.docx]

**Supplementary Table 2. The correlations between IBI and other inflammatory factors**

| IBI | r | *P* |
| --- | --- | --- |
| Neutrophil, 10^9/L | 0.488 | **<.001** |
| Lymphocyte, 10^9/L | -0.515 | **<.001** |
| NLR | 0.726 | **<.001** |
| CRP, mg/L | 0.838 | **<.001** |

CRP = C-reactive protein; NLR = neutrophil-to-lymphocyte ratio; IBI = inflammatory burden index.
