# Supplementary material for: Predictive value of IBI for acute kidney injury with contrast after PCI in patients with ST-segment elevation myocardial infarction
Source: Front Cardiovasc Med. 2025 Mar 20;12:1562731. doi: 10.3389/fcvm.2025.1562731 (PMC11965358; doi:10.3389/fcvm.2025.1562731)
Supplement: Supplementary file 3 [file Table3.docx]

**Supplementary Table 3. Univariate regression analysis**

|  | OR (95%CI) | *P* |
| --- | --- | --- |
| Age, years | 1.017 (0.998 ~ 1.037) | 0.078 |
| Female, n (%) | 1.307 (0.793 ~ 2.157) | 0.294 |
| Heart rate, bpm | 1.010 (0.994 ~ 1.026) | 0.229 |
| SBP, mmHg | 0.999 (0.987 ~ 1.011) | 0.875 |
| DBP, mmHg | 1.003 (0.986 ~ 1.020) | 0.748 |
| BMI, kg/m^2^ | 1.052 (0.991 ~ 1.117) | 0.097 |
| Smoking, n (%) | 1.043 (0.649 ~ 1.675) | 0.863 |
| Hypertension, n (%) | 0.965 (0.599 ~ 1.555) | 0.884 |
| Diabetes mellitus, n (%) | 1.796 (1.093 ~ 2.953) | **0.021** |
| CKD, n (%) | 0.422 (0.055 ~ 3.213) | 0.405 |
| MI, n (%) | 1.354 (0.548 ~ 3.342) | 0.512 |
| IBI > 18.89 | 3.851 (2.218 ~ 6.687) | **<.001** |
| White blood cell,10^9/L | 1.051 (0.980 ~ 1.127) | 0.167 |
| Neutrophil,10^9/L | 1.052 (0.999 ~ 1.108) | 0.056 |
| Lymphocyte,10^9/L | 0.495 (0.341 ~ 0.720) | **<.001** |
| Hemoglobin, g/L | 0.998 (0.984 ~ 1.012) | 0.790 |
| Platelet,10^9/L | 0.997 (0.993 ~ 1.001) | 0.146 |
| NLR | 1.055 (1.024 ~ 1.088) | **<.001** |
| Serum creatinine, μmol/L | 1.002 (0.990 ~ 1.013) | 0.774 |
| eGFR, mL/min/1.73 m^2^ | 0.987 (0.977 ~ 0.998) | **0.018** |
| CRP, mg/L | 1.095 (1.036 ~ 1.157) | **0.001** |
| TnI, ng/mL | 1.012 (0.998 ~ 1.027) | 0.099 |
| NT-proBNP, pg/mL | 1.584 (1.303 ~ 1.925) | **<.001** |
| FBG, mmol/L | 1.143 (1.070 ~ 1.222) | **<.001** |
| Total cholesterol, mmol/L | 1.115 (0.882 ~ 1.411) | 0.362 |
| Triglycerides, mmol/L | 0.759 (0.549 ~ 1.048) | 0.094 |
| HDL-C, mmol/L | 1.687 (0.640 ~ 4.448) | 0.290 |
| LDL-C, mmol/L | 1.146 (0.876 ~ 1.499) | 0.320 |
| Duration of operation, min | 1.005 (0.994 ~ 1.017) | 0.341 |
| Contrast agent, mL | 1.004 (0.998 ~ 1.010) | 0.152 |
| IABP, n (%) | 2.367 (0.842 ~ 6.654) | 0.102 |
| LVEF, % | 0.950 (0.919 ~ 0.982) | **0.002** |
| Killip class>2, n (%) | 2.089 (1.181 ~ 3.696) | **0.011** |
| IRA-LAD, n(%) | 1.667 (1.029 ~ 2.701) | **0.038** |
| IRA-LCX, n(%) | 1.195 (0.566 ~ 2.523) | 0.641 |
| IRA-RCA, n(%) | 0.542 (0.322 ~ 0.913) | **0.021** |
| ACEI/ARB, n(%) | 1.433 (0.890 ~ 2.309) | 0.139 |
| β-blockers, n(%) | 0.982 (0.497 ~ 1.939) | 0.958 |
| Nitrates, n(%) | 0.716 (0.432 ~ 1.185) | 0.194 |
| Heparin, n(%) | 0.928 (0.500 ~ 1.722) | 0.812 |
| Diuretics, n(%) | 1.503 (0.919 ~ 2.455) | 0.104 |

BMI = body Mass Index; IRA = infarct-related arteries; IABP = intra-aortic balloon pump; LVEF = left ventricular ejection fraction; CKD = chronic kidney disease; SBP = systolic blood pressure; DBP = diastolic blood pressure; IBI = inflammatory burden index; MI = myocardial infarction; LAD = left anterior descending; LCX = left circumflex artery; RCA = right coronary artery; LM = left main; ACEI = angiotensin-converting-enzyme inhibitor; ARB = angiotensin II receptor blocker; HDL-C = high-density leptin cholesterol; LDL-C = low-density leptin cholesterol; CRP = C-reactive protein; NLR = neutrophil-to-lymphocyte ratio; TnI = troponin I; NT-proBNP = N-terminal pro-B-type natriuretic peptide; FBG = fasting blood glucose; CI-AKI = contrast-induced acute kidney injury.
