# Supplementary material for: Predictive value of IBI for acute kidney injury with contrast after PCI in patients with ST-segment elevation myocardial infarction
Source: Front Cardiovasc Med. 2025 Mar 20;12:1562731. doi: 10.3389/fcvm.2025.1562731 (PMC11965358; doi:10.3389/fcvm.2025.1562731)
Supplement: Supplementary file 4 [file Table4.docx]

**Supplementary Table 4. ROC Curve of models for CI-AKI**

|  | AUC | 95% CI | *P* | Sensitivity | Specificity |
| --- | --- | --- | --- | --- | --- |
| NT-proBNP+FBG | 0.710 | 0.656 ~ 0.764 | <0.001 | 0.846 | 0.511 |
| NT-proBNP+FBG+IBI | 0.737 | 0.684 ~ 0.790 | <0.001 | 0.897 | 0.475 |

IBI = inflammatory burden index; NT-proBNP = N-terminal pro-B-type natriuretic peptide; FBG = fasting blood glucose; CI-AKI = contrast-induced acute kidney injury.
